# GLI1-Altered Mesenchymal Tumor with ACTB Fusion and Somatostatin Receptor 2A (SSTR2A) Immunopositivity

**DOI:** 10.7759/cureus.74325

**Published:** 2024-11-23

**Authors:** John Grove, Daniel L Geisler, Rana Naous

**Affiliations:** 1 Pathology and Laboratory Medicine, University of Pittsburgh Medical Center, Pittsburgh, USA; 2 Department of Pathology and Laboratory Medicine, University of Pittsburgh Medical Center East, Monroeville, USA; 3 Pathology, University of Pittsburgh Medical Center, Pittsburgh, USA

**Keywords:** actb, fusion, gli1, mesenchymal tumor, sstr2a

## Abstract

Glioma-associated oncogene (*GLI1)*-altered mesenchymal tumors are a newly described entity of neoplasms with very few case reports published in the literature. *GLI1*-altered neoplasms have a moderate degree of variability as they are seen in a broad range of anatomic sites and amongst people of all ages. A common feature that most *GLI1*-altered tumors share is the histologic makeup of monomorphic ovoid cells organized in distinct nests and an arborizing vascular blood supply. While previously thought to be benign entities, more recent studies have highlighted metastatic potential within this group of tumors, further emphasizing the variability within this class of disease and the necessity to further understand the behavior of these tumors. Here, we report a 36-year-old male presenting with a palpable mass centered within the right gluteus maximus muscle. The mass was excised and measured 6.3 cm in the greatest dimension, with histologic findings and molecular workup confirming the diagnosis of a *GLI1*-altered mesenchymal tumor with beta-acting gene (*ACTB)* fusion. Interestingly, the tumor stained positive for somatostatin receptor 2A (SSTR2A), a stain that has yet to be elucidated as a potential diagnostic tool for this rare entity.

## Introduction

Glioma-associated oncogene (*GLI1)*-altered mesenchymal tumors are a newly described class of tumors with molecular alterations of* *the* GLI1* gene, usually fusions or amplifications [1]. Common molecular fusion partners seen are *GLI1::ACTB*, *GLI1::PTCH1* or *GLI1::MALAT1* [2]. The GLI1 protein is a transcription factor activated by the sonic hedgehog signal transduction cascade and plays an important role in stem cell proliferation [3]. Pathologic activation of the hedgehog signaling pathway has been documented in many tumors, such as basal cell carcinoma, gliomas, and breast carcinomas [4]. *GLI1*-altered mesenchymal tumors have been reported in a variety of anatomic locations, with tumors in the soft tissue, head and neck, trunk, and extremities being the most common [4,5]. Histologically, these tumors appear to be multinodular with a distinctive nested architecture accompanied by a rich arborizing vascular network [4]. On high power, a population of monomorphic cells with uniform oval nuclei and variably eosinophilic to clear cytoplasm can be appreciated [4,5]. The degree of mitotic activity is variable. *GLI1*-altered mesenchymal tumors typically stain positive for CD56 and S100 [4,6]. Other useful markers include SOX10, CD31, CD34, synaptophysin, CD99 and desmin. Somatostatin receptor 2A (SSTR2A) immunopositivity has not been reported to date in *GLI1*-altered mesenchymal tumors. Herein, we report a *GLI1*-altered mesenchymal tumor with ACTB fusion and unique immunohistochemical staining for SSTR2A and will discuss how this staining can impact the differential diagnosis of *GLI1*-altered mesenchymal tumors.

## Case presentation

**Figure 1:**
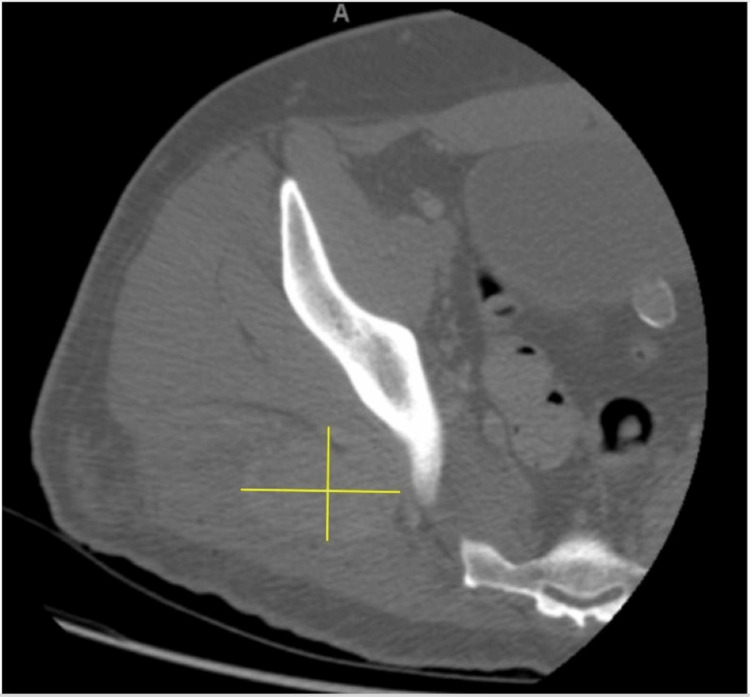
CT right lower extremity with contrast CT right lower extremity with contrast showing a heterogeneously enhancing soft tissue mass centered in the right gluteus maximus muscle measuring 6.3 x 4.0 cm (marked).

A 36-year-old male with a medical history of essential hypertension, viral cardiomyopathy, Chiari malformation, and coronary artery disease presented with a chief complaint of a right buttock mass. The mass was non-tender to palpation. A complete blood count (CBC) and a comprehensive metabolic panel were performed and were non-contributory. CT of the right lower extremity with contrast showed a heterogeneously enhancing soft tissue mass centered in the right gluteus maximus muscle measuring 6.3 x 4.0 cm (Figure 1).

**Figure 2:**
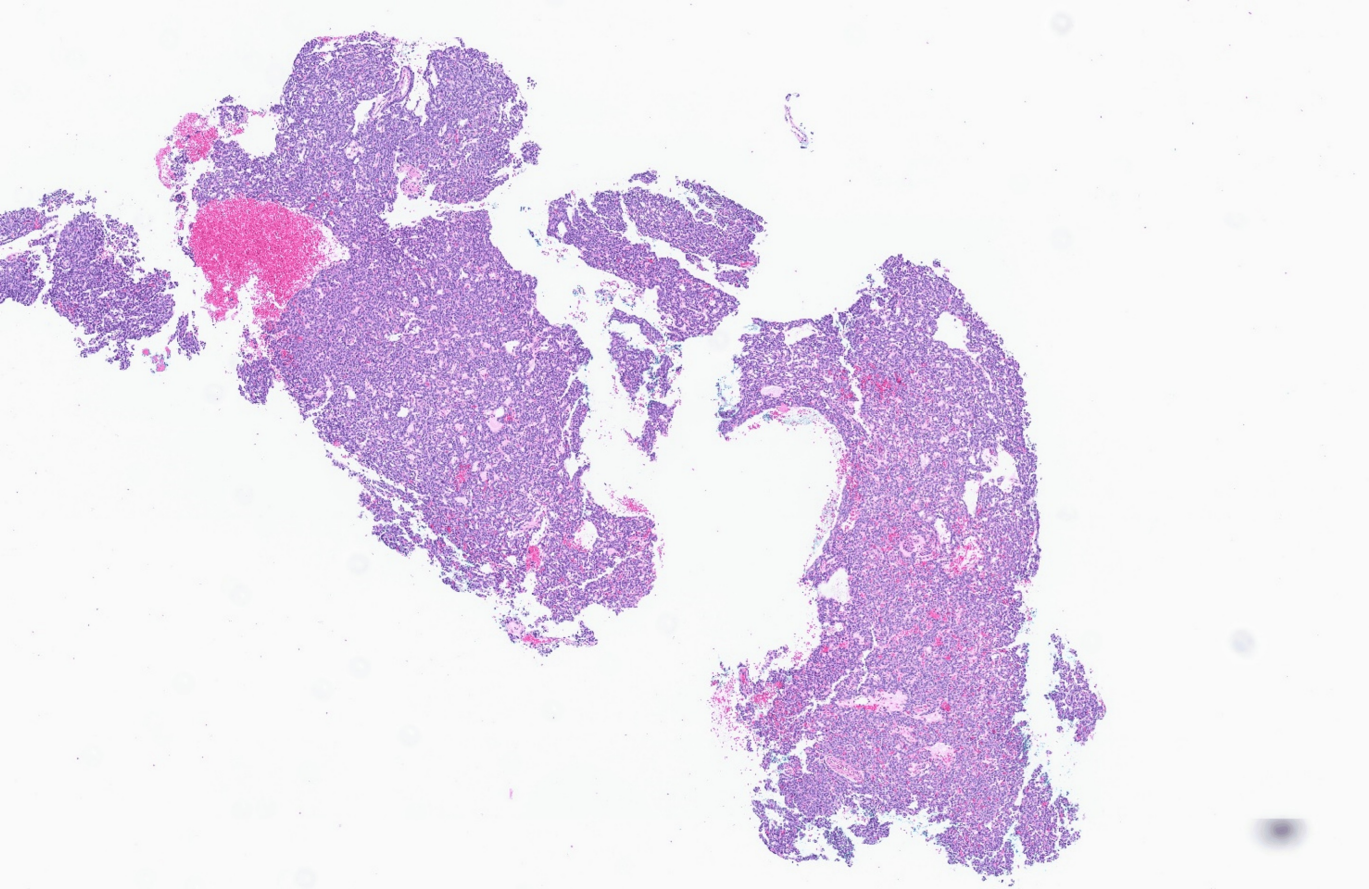
GLI1-altered mesenchymal tumor at low power magnification Glioma-associated oncogene (*GLI1*)-altered mesenchymal tumor at low power magnification demonstrating the nested and corded arrangement of the tumor cells (H&E, 4X). The tumor cells appear monomorphic and are set in a richly vascular storma.

**Figure 3:**
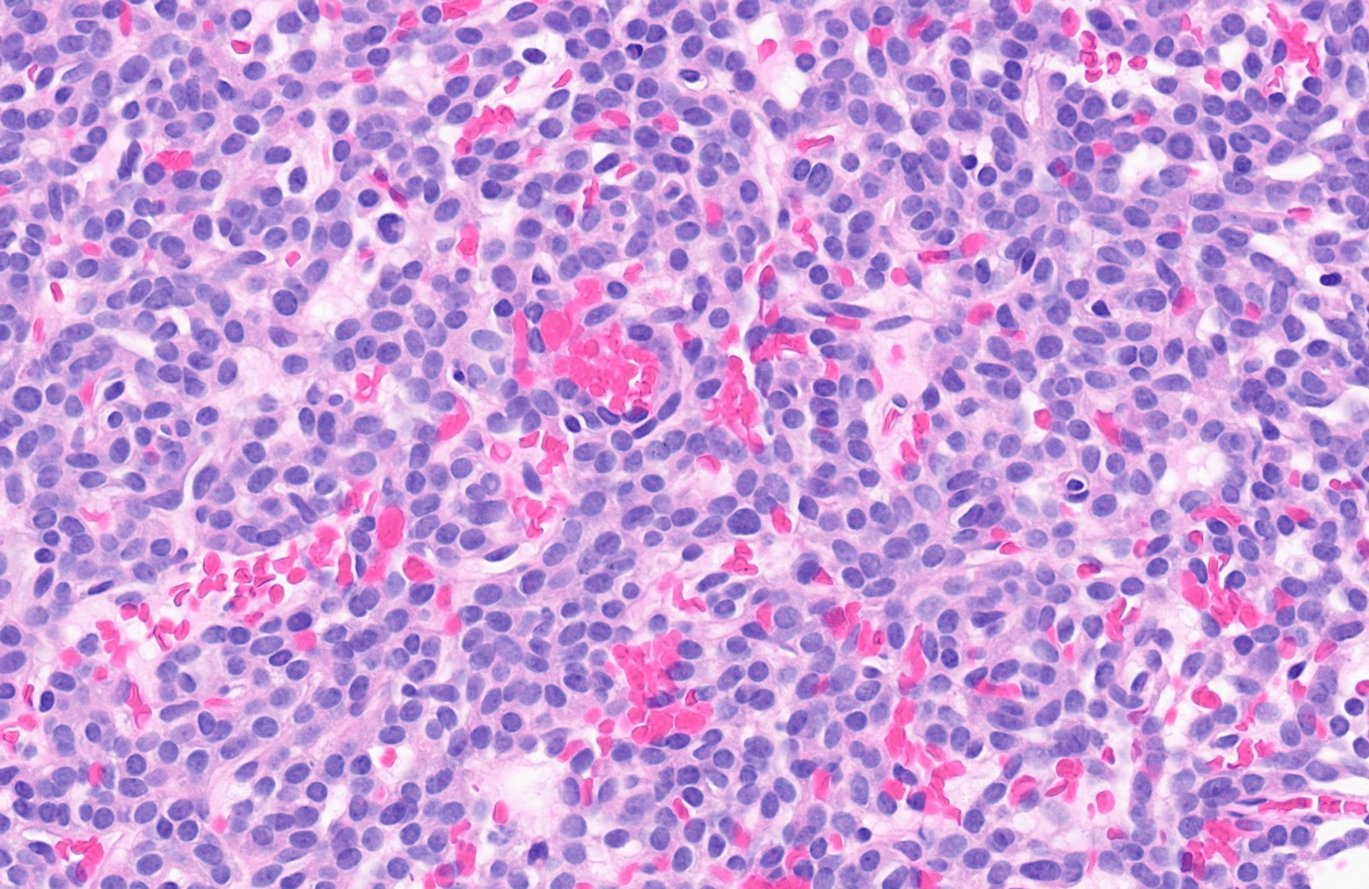
GLI1-altered mesenchymal tumor at high power magnification Glioma-associated oncogene (*GLI1*)-altered mesenchymal tumor at high power magnification demonstrating relatively monomorphic round to ovoid cells with mild atypia, fine chromatin, small nucleoli, and a moderate amount of eosinophilic cytoplasm (H&E, 40X). Note the nested arrangement of the tumor cells and the rich vascular background.

Biopsy of the mass showed nests and cords of round to ovoid cells with fine chromatin, occasional small nucleoli, intranuclear inclusions and a moderate amount of eosinophilic cytoplasm. There was mild to moderate nuclear atypia, and mitotic activity reached up to three mitoses per 10 high-power fields (Figures 2, 3).

**Figure 4:**
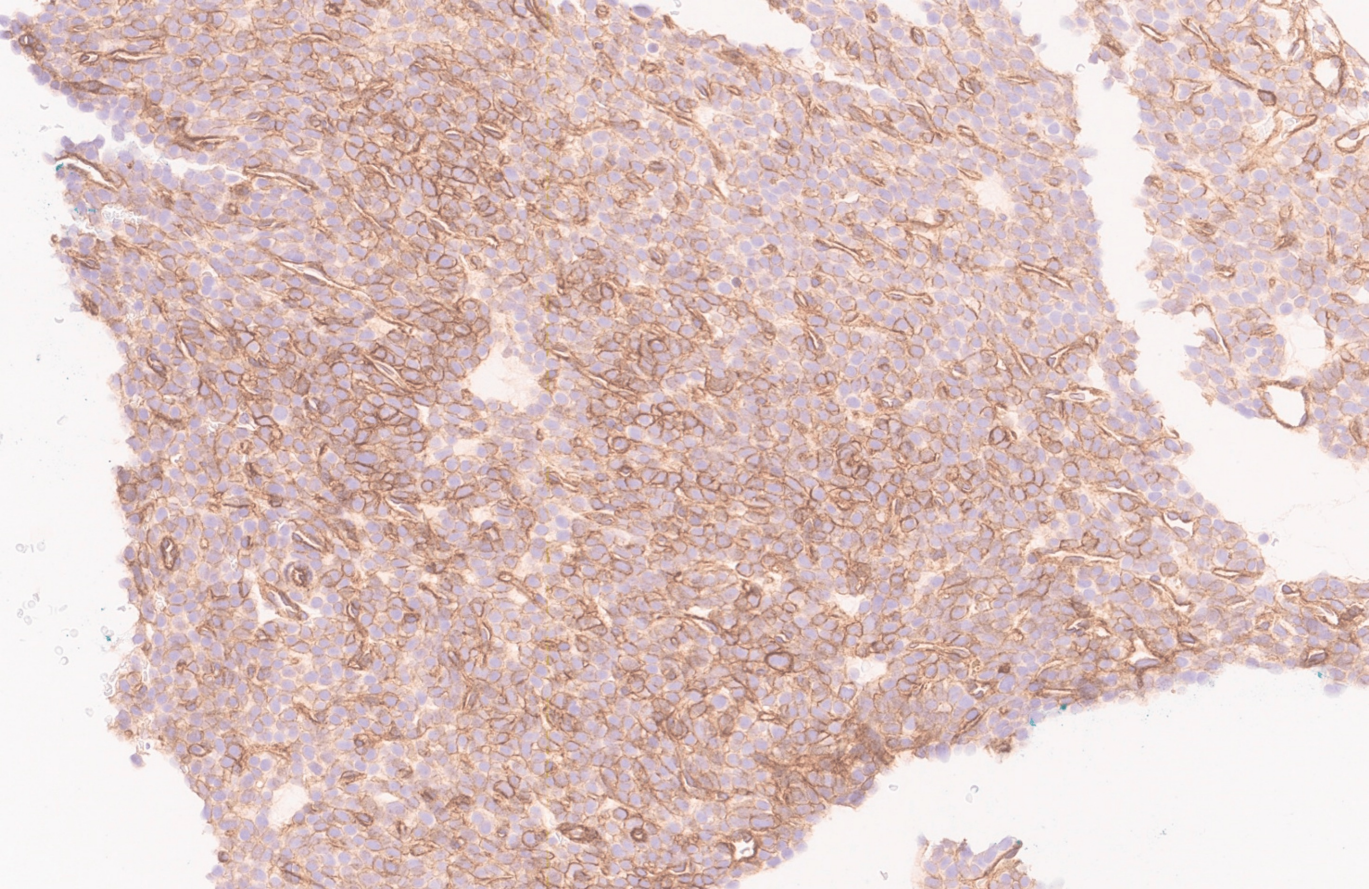
SMA immunostain image Smooth muscle antibody (SMA) immunostain showing cytoplasmic and membranous positivity in the tumor cells (SMA stain, 20X). The stain accentuates the membrane borders of the tumor cells.

The smooth muscle antibody (SMA) immunostain test result is shown in Figure 4.

**Figure 5:**
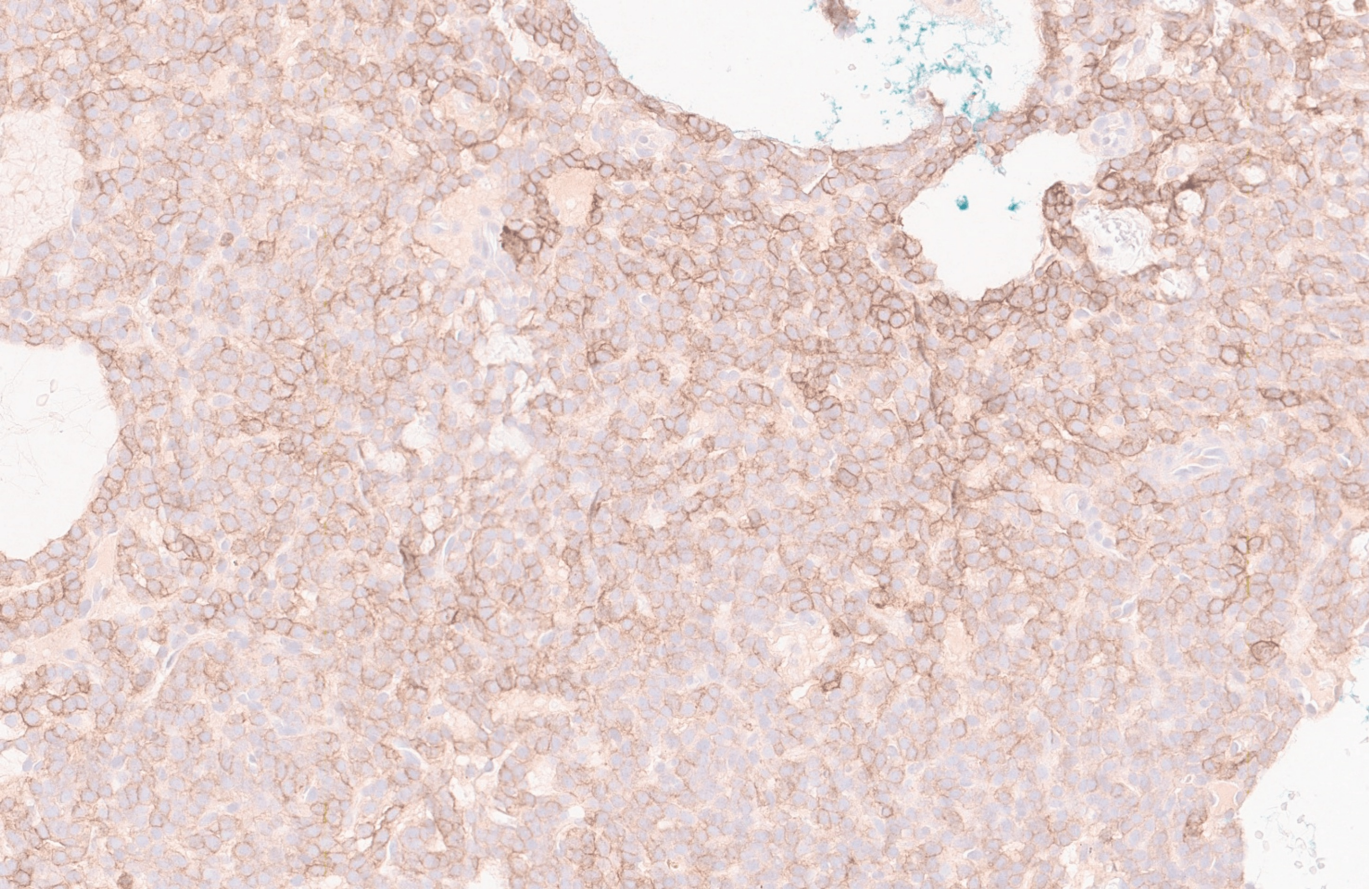
SSTR2A immunostain histochemistry Somatostatin receptor 2A (SSTR2A) immunostain positive in tumor cells (SSTR2A stain, 20X). The stain highlights the cytoplasmic borders within the tumor cells with variable staining intensity.

**Figure 6:**
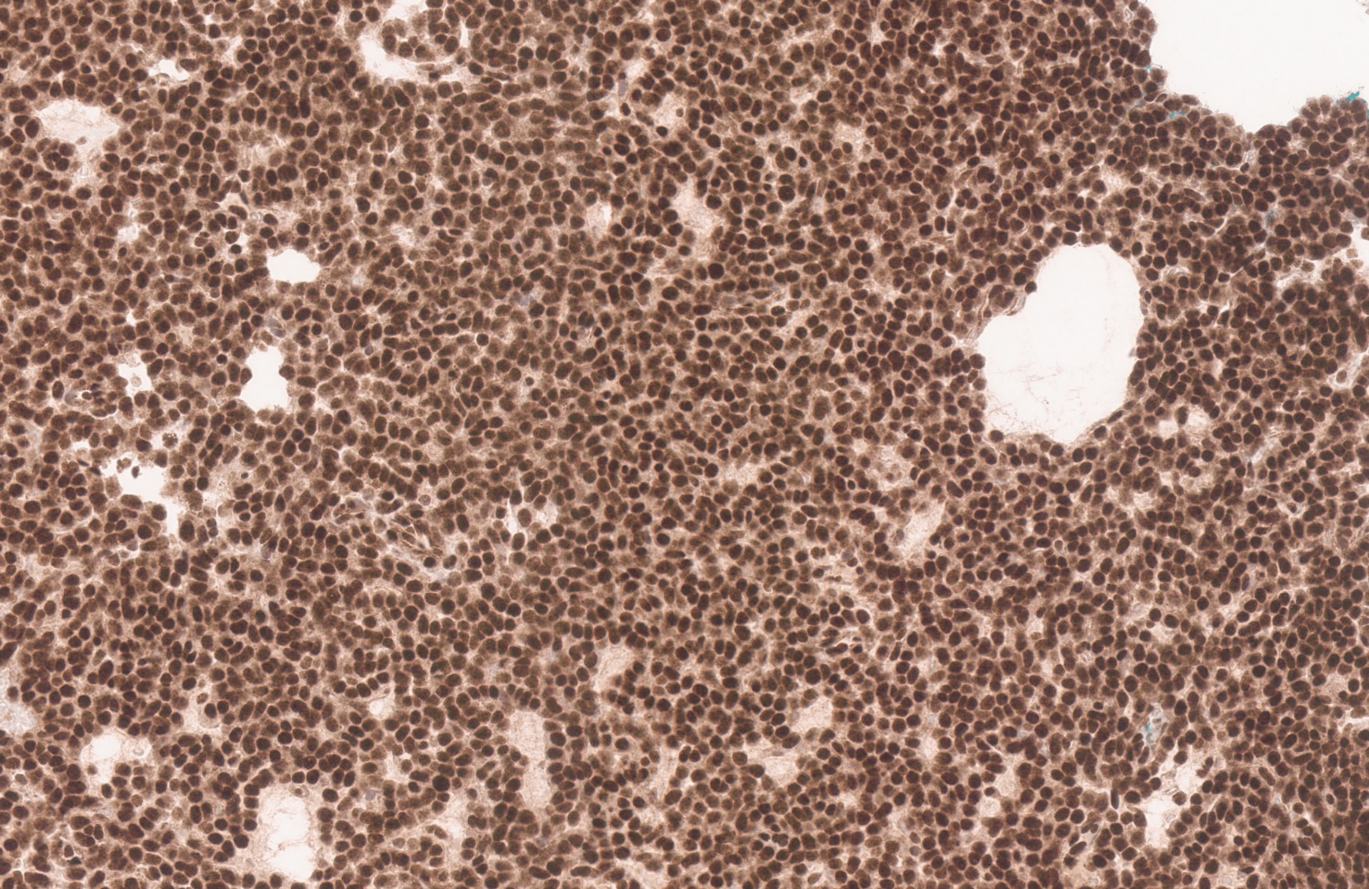
INI1 immunostain histochemistry Integrase Interactor 1 (INI1) immunostain retained in tumor cells (INI1 stain, 20X). The tumor cells demonstrate intense nuclear staining; thus confirming the presence of *SMARCB1* gene wild type at the molecular level.

SMA showed patchy immunopositivity in the tumor cells, while S100, SOX10, HMB45, CAM5.2, EMA, cytokeratin AE1/AE3, desmin, myogenin, CD34, ERG, CD31, HHV 8, INSM1, WT1, STAT6, LCA, CD21, ALK1, caldesmon, smooth muscle myosin heavy chain, Melan-A, CK5/6, p40, MUC4, NUT, GATA3, CD21, and CD23 immunostains were essentially negative. Additionally, the tumor cells showed positivity for SSTR2A (Figure 5) and integrase Interactor 1 (INI-1) retained nuclear expression (Figure 6).

Fluorescence in situ hybridization (FISH) studies for HMGA2 and SS18 (*SYT*) gene rearrangements were negative. Whole transcriptomic sequencing (RNA Seq) identified *ACTB::GLI1* gene fusion, corresponding to t(7;12)(p21-22;q13-15), ultimately supporting the diagnosis of *GLI1*-altered mesenchymal tumor with *ACTB* fusion.

## Discussion

*GLI1*-altered mesenchymal tumors are soft tissue tumors that arise in many regions of the body. This class of mesenchymal tumors comprises a wide array of molecular alterations, with the most common being *GLI1::ACTB*, *GLI1::PTCH1* or *GLI1::MALAT1* [2,4].

*GLI1*-altered mesenchymal tumors are a recently described emerging entity. In the limited available data, although a subset of cases has been reported to behave in an indolent fashion, recent literature has reported the possibility for local-regional recurrence, metastasis, and disease progression, even in cytologically bland nested tumors [5]. While the mitotic count is described as variable in the literature, with mitotic counts per high power field ranging from 0 to 10, studies have reported that a mitotic count of greater than or equal to four mitoses per 10 high power fields is associated with a worse prognosis [7].

The majority of case reports and published literature emphasize that CD56 and S100 immunostains are occasionally positive with this group of neoplasms [4,6]. Other immunohistochemical stains that are typically positive, at least focally, include CD34, synaptophysin, CD99, and desmin [4]. Additionally, GLI1 immunostains can be utilized as well, however caution must be taken as GLI1 gene amplification is seen in a number of sarcomas and carcinomas leading to GLI1 protein overexpression and positive immunostaining which may lead to potential diagnostic pitfalls [8].

During the workup of our case, a number of immunostains were ordered, including SSTR2A (somatostatin receptor 2A) to rule out a neuroendocrine neoplasm. We conducted a literature review and were not able to find any publication of SSTR2A immunohistochemical positivity in *GLI1*-altered mesenchymal tumors. As mentioned previously, *GLI1*-altered mesenchymal tumors occur in various anatomic locations, with the head and neck region being one of the more commonly reported sites of these tumors. SSTR2A is well-documented in other tumors in the head and neck regions such as ectopic meningeal tumors [9]. Thus, a *GLI1*-altered mesenchymal tumor should be included in the differential in any neoplasm in the head and neck that stains positively for SSTR2A.

Other differential diagnoses of *GLI1*-altered mesenchymal tumors include glomus tumor, myoepithelial tumor and epithelioid schwannoma. Our case was initially suspected to be a glomus tumor based on the relatively monomorphic and nested arrangement of the round-to-ovoid tumor cells with minimal atypia and patchy SMA staining. However, the focal rather than diffuse staining for SMA and the unique expression of SSTR2 were unusual features that argued against a glomus tumor. The absence of S100 and keratin staining in our case essentially excluded a myoepithelial tumor. Additionally, the negative S0X10 immunostain basically ruled out an epithelioid schwannoma. Ultimately, if a tumor is suspected to harbor a pathologic *GLI1* gene alteration, molecular analysis should be performed to ascertain the underlying genetic defect and prove the presence of *GLI1* gene fusion.

SSTR2A expression has been associated with improved clinical outcomes in solid tumors [10] and can act as a potential therapeutic target in certain tumors, including neuroendocrine neoplasms, olfactory neuroblastoma, and small-cell lung cancer [11]. SSTR2A immunostaining has been reported to be positive in multiple tumors other than meningiomas including pituitary adenoma [12], small intestine, pancreatic, and breast neuroendocrine tumors [13], small cell lung cancer [14], paraganglioma and pheochromocytoma [15], gastrointestinal stromal tumor [16], follicular dendritic cell sarcoma [17], olfactory neuroblastoma [18], lymphoepithelioma-like carcinoma [19], and nasopharyngeal Epstein-Barr virus-associated carcinoma [20].

The novel SSTR2A immunopositivity in our case is of uncertain significance at this point in time as more cases of *GLI1*-altered mesenchymal tumors will need to be examined for this protein expression to draw conclusions about its potential therapeutic or clinical outcome.

Given their potential for metastasis and disease progression, the term "*GLI1*-altered mesenchymal tumor with malignant potential" has been proposed by some authors, with indications for complete surgical resection with negative margins and close clinical surveillance, akin to that for sarcoma management [2]. In our case, the patient is planned for complete excision with wide negative margins.

## Conclusions

Herein, we report a case of a *GLI1*-altered mesenchymal tumor in a 36-year-old male with novel immunohistochemical staining for SSTR2A. As our knowledge of this recently described neoplasm continues to develop, it is important to further the understanding of its pathogenesis and different diagnostic tools to arrive at the correct diagnosis. The presence of SSTR2A staining in *GLI1*-altered mesenchymal tumors is a diagnostic pitfall that may lead to an erroneous diagnosis, especially in tumors arising in head and neck locations. Awareness of such phenomenon, along with having a broad differential diagnosis and performing thorough immunohistochemical and molecular ancillary testing, are critical diagnostic tools that can help avoid potential diagnostic pitfalls and aid in reaching the correct diagnosis. 
